# A SGLT2 Inhibitor Dapagliflozin Alleviates Diabetic Cardiomyopathy by Suppressing High Glucose-Induced Oxidative Stress *in vivo* and *in vitro*

**DOI:** 10.3389/fphar.2021.708177

**Published:** 2021-07-12

**Authors:** Yu-jie Xing, Biao-hu Liu, Shu-jun Wan, Yi Cheng, Si-min Zhou, Yue Sun, Xin-ming Yao, Qiang Hua, Xiang-jian Meng, Jin-han Cheng, Min Zhong, Yan Zhang, Kun Lv, Xiang Kong

**Affiliations:** ^1^ Key Laboratory of Non-coding RNA Transformation Research of Anhui Higher Education Institution, Wannan Medical College, Wuhu, China; ^2^ Department of Endocrinology, The First Aflliated Hospital of Wannan Medical College, Yijishan Hospital, Wuhu, China; ^3^ Department of Ultrasound Medicine, The First Aflliated Hospital of Wannan Medical College, Yijishan Hospital, Wuhu, China; ^4^ Central Laboratory of Yijishan Hospital, Wuhu, China

**Keywords:** diabetic cardiomyopathy, dapagliflozin, oxidative stress, nicotinamide adenine dinucleotide phosphate oxidase, myocardial apoptosis

## Abstract

Diabetic cardiomyopathy (DCM) is a serious complication of diabetes mellitus (DM). One of the hallmarks of the DCM is enhanced oxidative stress in myocardium. The aim of this study was to research the underlying mechanisms involved in the effects of dapagliflozin (Dap) on myocardial oxidative stress both in streptozotocin-induced DCM rats and rat embryonic cardiac myoblasts H9C2 cells exposed to high glucose (33.0 mM). In *in vivo* studies, diabetic rats were given Dap (1 mg/ kg/ day) by gavage for eight weeks. Dap treatment obviously ameliorated cardiac dysfunction, and improved myocardial fibrosis, apoptosis and oxidase stress. In *in vitro* studies, Dap also attenuated the enhanced levels of reactive oxygen species and cell death in H9C2 cells incubated with high glucose. Mechanically, Dap administration remarkably reduced the expression of membrane-bound nicotinamide adenine dinucleotide phosphate (NADPH) oxidase subunits gp91phox and p22phox, suppressed the p67phox subunit translocation to membrane, and decreased the compensatory elevated copper, zinc superoxide dismutase (Cu/Zn-SOD) protein expression and total SOD activity both *in vivo* and *in vitro*. Collectively, our results indicated that Dap protects cardiac myocytes from damage caused by hyperglycemia through suppressing NADPH oxidase-mediated oxidative stress.

## Introduction

Diabetes mellitus (DM) with hyperglycemia as the main feature has become a global public health problem (Wild, 2004). Diabetes-related cardiovascular complications are major factors in the population mortality and morbidity of diabetes (Melin et al., 2018). Diabetic cardiomyopathy (DCM) is marked by myocardial left ventricular dysfunction without obvious atherosclerosis and coronary artery disease (Dillmann, 2019). Although the pathogenesis and clinical features of DCM have been fully elucidated in the past decade, no consensus has been reached regarding the most effective preventive or therapeutic approaches to treat this disease.

Sodium glucose cotransporter-2 (SGLT2) inhibitors are anti-diabetic drugs that can reduce blood glucose of diabetic patients through inhibiting renal glucose reabsorption (Heerspink et al., 2016). SGLT2 inhibitors have shown superiority in cardiovascular diseases than other hypoglycemic agents (Ren and Zhang, 2018). Dapagliflozin (Dap), a highly selective SGLT2 inhibitor, is used as an effective treatment against type 2 DM and prevents the development of diabetic nephropathy (Wheeler et al., 2020; Wheeler et al., 2021). Rising evidences have proved that Dap declines the rate of both cardiovascular death and heart failure hospitalization (McMurray et al., 2019; Wiviott et al., 2019). Recently, Dap has been proved to reduce myocardial NLRP3 inflammasome activation, and to improve the aggravation of left ventricular ejection fraction in ob/ob type 2 DM mice (Ye et al., 2017; Chen et al., 2020). Arow et al. (2020) reported that Dap decreases myocardial inflammation and reactive oxygen species (ROS) production in db/db type 2 DM mice and isolated cardiomyocytes. These data indicate that Dap is a possible therapeutic agent in the treatment of DCM through anti-inflammation and anti-oxidative stress. However, the molecular mechanisms of Dap treatment resulting in the decrease of myocardial ROS levels has not been completely elucidated.

Oxidative stress defined as an increase of ROS content, is resulted from the imbalance between the ROS generation and the antioxidant defense system and has a close relationship with the progression of cardiovascular diseases (Wold et al., 2005). NADPH oxidases (NOXs) are key enzymes that produce ROS and up-regulated in the heart of DCM (Hansen et al., 2018; Lu et al., 2020). Little evidence has suggested whether Dap ameliorates DCM by restraining the NADPH oxidases and/or regulating the antioxidant enzymes such as superoxide dismutase (SOD), glutathione peroxidase (GPx) and catalase (CAT). In light of the knowledge gap in the field, the present study was designed to clarify the mechanisms of Dap on the myocardial oxidative stress both in streptozotocin (STZ)-induced DCM rats and H9C2 cells exposed to high glucose.

## Materials and Methods

### Animals Studies

7-week-old male Sprague-Dawley (SD) rats were obtained from the Experimental Animal Center of Qinglongshan (Nanjing, China), and allowed to acclimatize for 1 week in their cages before experiments. All rats can freely have standard chow and water with a 12 h light–dark cycle in a controlled temperature of 24°C. Meanwhile, all experimental protocols were approved by the Animal Ethics Committee of Yijishan Hospital.

After 12 h of fasting, STZ (freshly dissolved in 0.1 M citrate buffer, pH 4.5) was given to the rats by intraperitoneal injection at a single dose of 60 mg/ kg. Fasting blood glucose (FBS) was gauged by a glucometer (One Touch Ultra, United States). Rats with FBS level >16.7 mM at 3 days after STZ injection were considered as diabetic model. The rats of control and hyperglycemic group were randomly divided into four groups (n = 7 each group) as follows: the untreated control group (Con), the Dap-treated control group (Con + Dap), the untreated STZ-induced diabetic group (STZ) as well as the Dap-treated diabetic group (STZ + Dap). Dap was provided by AstraZeneca Pharmaceutical Co. Ltd. Rats in the two treatment groups were orally administrated with Dap dissolved in pure water (average 1 mg/ kg/ day) for 8 weeks. Dap dosage was selected on the basis of previous studies, and 1 mg/ kg Dap has the sufficient *in vivo* cardioprotective effects (Tanajak, 2018; Gong et al., 2021). Body weight was recorded every week. Non-fasting blood glucose levels were measured at 9 AM every 2 weeks.

### Echocardiographic Evaluation

Two days before the end of the study period, echocardiographic measurement was done. 1.5% isoflurane was used to anesthetize rats. Left ventricular (LV) ejection fraction was obtained by M-mode echocardiography. We used 17.5 MHz liner array transducer system and then measured at least five cardiac cycles on the M-mode tracings. All LV tracings were manually measured by the same observer.

### Rats Myocardial Masson and TUNEL Staining

When the study was ended, all rats were fasted overnight. Then they were anaesthetized by sodium pentobarbital. The dose was 30 mg/ kg by intraperitoneal injection. Hearts were rapidly removed, washed, dried and weighed. Part of the LV tissues were fixed in 10% neutral buffered formalin for histological analysis later. A portion of LV tissues were quickly frozen in OCT embedding medium (Sakura Finetek, United States). We used them for superoxide anion detection. The remaining heart tissues were immediately stored at–80°C. Sections (5 μm thick) of paraﬃn-embedded rat heart tissues were used for Masson trichrome and TUNEL staining according to standard protocols.

### Isolation of RNA and qRT-PCR in Rat Myocardium

The heart tissues were added suitable TRIzol reagent (Invitrogen). Hifair**®** III 1st Strand cDNA Synthesis Kit (11139ES60, Yeasen, China) was used to synthesize cDNA. Quantitative real-time PCR (qRT-PCR) was performed with Hieff**®** qPCR SYBR Green Master Mix (No Rox) (11201ES08, Yeasen, China) according to the instruction. The mRNA expression of GAPDH was used as a reference. The primer sequences used for sodium-hydrogen exchanger 1 (NHE-1) and GAPDH were as follows: NHE-1, forward 5′-ACA​TTC​AAC​AGT​GGA​GTG ACT-3′ and reverse 5′-TGGCAGGGAAGA TTCAAAGG-3′. GAPDH, forward 5′-TGC​ACC​ACC​AAC​TGC​TTA​GC-3′ and reverse 5′-GCC​CCA​CGG​CCA​TCA-3′.

### Measurement of Superoxide Anion Generation in Rat Myocardium

Dihydroethidium (DHE) specifically reacts with superoxide to form ethidium, which stains DNA, showing a red fluorescence. 6 μm sections of rat LV tissues were taken in a freezing microtome. Then, the slides were incubated in 5 µM DHE (Beyotime Biotechnology Inc., China) in PBS at 37°C for 30 min in a damp and dark container. The images of thidium fluorescent were obtained with a fluorescence microscope (Leica Microsystems, Germany).

### Detection of Malondialdehyde Level in Rat Myocardium

The level of MDA in rat LV tissues was quantified using a Lipid Peroxidation MDA Assay Kit (Beyotime Biotechnology Inc., China). Briefly, the LV tissues were lysed in RIPA lysis buffer. Then the supernatant was collected after centrifuging at 10,000 × g for 10 min and was used to quantify MDA concentration according to the thiobarbituric acid (TBA) method. The absorbance of the product of MDA-TBA was finally determined at 535 nm using the Multi-Mode Microplate Reader (Bio Tek Instruments Inc., United States).

### Cell Culture and Processing

Rat embryonic cardiac myoblasts H9C2 cells were purchased from the Shanghai Institute of Biochemistry and Cell Biology (Shanghai, China) and maintained at 37°C (5% CO_2_ and 95% O_2_) in Dulbecco's modified Eagle’s medium (DMEM, Gibco) supplemented with 10% fetal bovine serum (FBS) (LONESERA, Uruguay in South America). DAP was dissolved in pure water and used at a final concentration of 10 µM. The dosage of Dap was selected based on previous studies (Huang et al., 2019; Arow et al., 2020), and our preliminary experiment showed that 10 µM Dap has the sufficient *in vitro* antioxidant capacity.

H9C2 cells were put in suitable plates and treated as follows for 36 h, namely the normal glucose (5.5 mM, Con) group, the normal glucose with 10 µM Dap (Con + Dap) group, the mannitol (33.0 mM, Man) group, the high glucose (33.0 mM, HG) group, and the high glucose with 10 µM Dap (HG + Dap) group. The Man group was used to estimate the influence of osmotic pressure.

### Calcein Acetoxymethyl Ester/Propidium Iodide Staining in H9C2 Cells

H9C2 cells were cultured in 96-well plates. Given different treatments, they were cultured in assay buffer mixed with 1 µM calcein acetoxymethyl ester and 1 µM PI per well at 37°C for 30 min according to the introduction of Calcein-AM/PI Cell Viability/Cytotoxicity Assay Kit (Beyotime Biotechnology Inc., China). Green fluorescence by Calcein-AM indicates living cells. Red fluorescence by PI represents dead cells. The images were acquired with a fluorescence microscope (Leica Microsystems, Germany). The percentage of positive cells was counted with Image J software and each group had more than 10,000 cells.

### Measurement of Hydrogen Peroxide Generation in H9C2 Cells

Intracellular generation of hydrogen peroxide was assayed using fluorescent probe 2′,7′-dichlorodihydrofluorescin diacetate (DCFH-DA, Beyotime Biotechnology Inc., China). If there exists hydrogen peroxide, DCFH-DA is oxidized into the fluorescent DCF, which can be detected by flow cytometry and fluorescence microscope.

H9C2 cells were placed in six-well plates. After various treatments for the indicated time, the cells with 10 µM DCFH-DA were put in the dark with 37°C for at least 30 min. Then, the cells were rinsed with DMEM medium without fetal bovine serum. The images were photographed by a fluorescence microscope (Leica Microsystems, Germany) using 488 nm excitation and 525 nm emission wavelength. Furthermore, cellular fluorescence was detected by flow cytometry (Beckman Coulter, United States) analysis and analyzed with FlowJo software.

### Measurement of Superoxide Production in H9C2 Cells

As mentioned before, H9C2 cells were plated in six-well plates. After treatments for the indicated time, the cells were incubated with 5 µM DHE and put in the dark with 37°C for 30 min. Fluorescent images were obtained using a fluorescence microscope (Leica Microsystems, Germany).

Intracellular superoxide level was measured using the Superoxide Assay Kit (Beyotime Biotechnology Inc., China) in the H9C2 cells after different treatments (Wang et al., 2018; Zhang et al., 2019). The cells were incubated with the superoxide test solution at 37°C for 3 min. After that, the absorbance was finally assessed at 450 nm using the Multi-Mode Microplate Reader (Bio Tek Instruments Inc., United States).

### Detection of Total SOD Activity in Rat Myocardium and H9C2 Cells

Total SOD activity was analyzed by a WST-8 method according to the instructions (Beyotime Biotechnology Inc., China). Briefly, 20 μL supernatant obtained from the rat LV tissues or H9C2 cells was mixed with 160 μL working solution of WST-8/enzyme. Next, add 20 μL reaction starter working solution in each well and incubate for 30 min at 37°C. Measure absorbance at 450 nm using the Multi-Mode Microplate Reader (Bio Tek Instruments Inc., United States).

### Western Blot Analysis

The samples of rat LV tissues or H9C2 cells were lysed in RIPA lysis buffer and centrifuged at 10,000 × g for 10 min at 4°C. Cytoplasm and membrane proteins were isolated by MinuteTM Plasma Membrane Protein Isolation and Cell Fractionation Kit (Invent Biotechnologies, United States).

As described in our previous studies (Kong et al., 2015a; Kong et al., 2015b), equal protein (30 or 40 μg) of rat LV tissues or H9C2 cells was separated by SDS-PAGE, transferred to a nitrocellulose membrane (PALL BioTrace, China) and then blocked with 5% skim milk. After that, the membrane was put in primary antibody overnight and appropriated secondary antibodies for 2 h. Antibodies against caspase 3, gp91phox, Na, K-ATPase, Cu/Zn-SOD, Mn-SOD, and CAT were purchased from ABclonal Technology Inc. (China). Antibodies against p22phox, p67phox, and GPx were bought from Santa Cruz Inc (United States). The total protein reference was β-actin. Meanwhile, the reference of membrane protein was Na, K-ATPase. Results were assessed by densitometry using Image J software.

### Statistical Analysis

Data were presented as mean ± standard deviation (S.D.). One-way analysis of variance followed by the Newman-Keuls test was used to compare the difference among different groups. Significance was set at a value of *p* < 0.05.

## Results

### Effects of Dap on Body Weight, Blood Glucose and Cardiac Function in STZ-Induced Diabetic Rats

**FIGURE 1:**
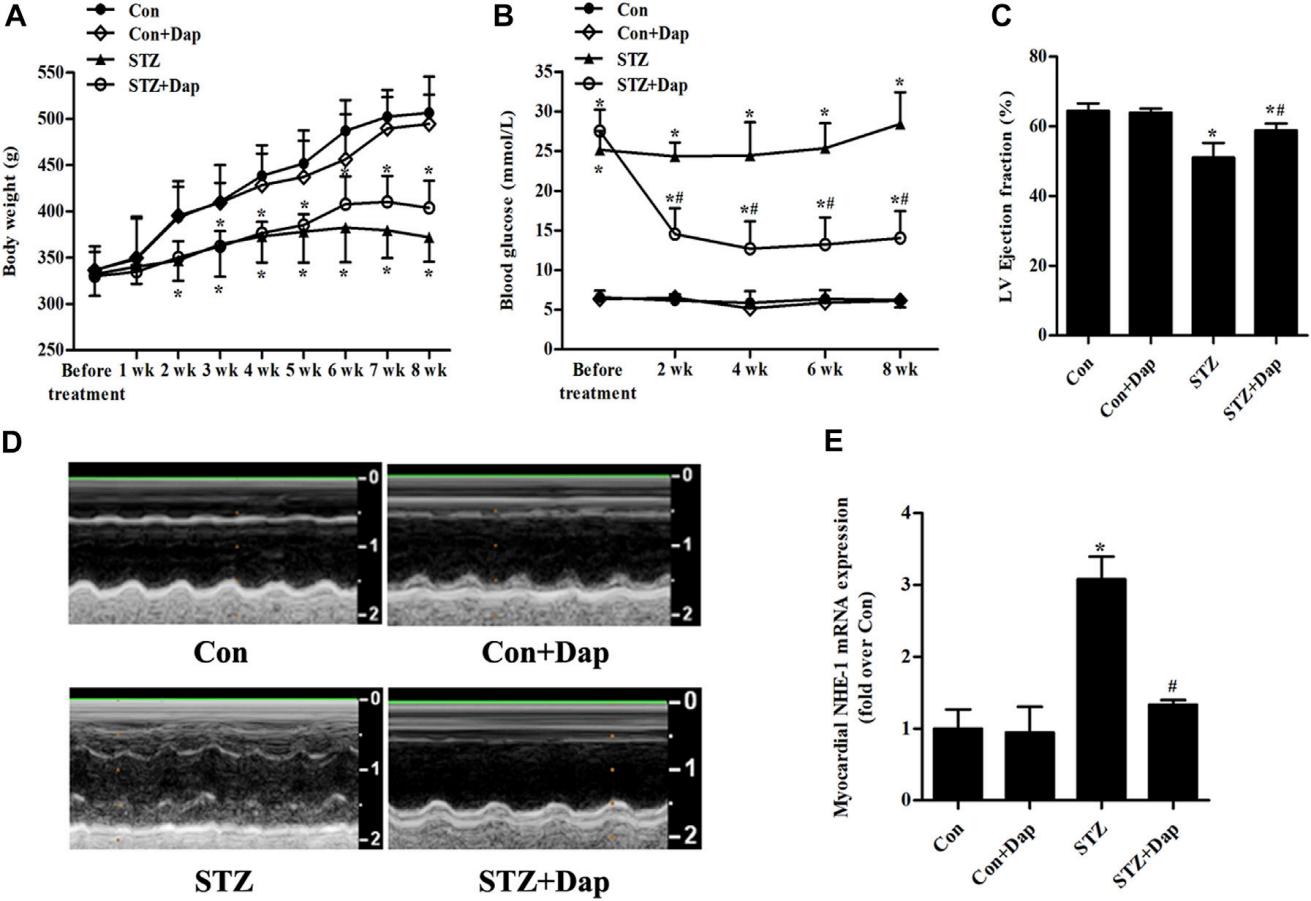
Effects of dapagliflozin (DAP) on body weight, non-fasting blood glucose and left ventricular (LV) dysfunction in diabetic rats. Control (Con + Dap) and STZ-induced diabetic rats (STZ + Dap) were administrated Dap by gavage at the daily dose of 1 mg/ kg for 8 weeks **(A)** Body weight was recorded weekly **(B)** Non-fasting blood glucose was measured at 9 AM on a bi-weekly basis **(C)** Echocardiography was used to measure LV ejection fraction **(D)** LV echocardiographic representative images from each group. *n* = 7 **(E)** The mRNA expression of myocardial NHE-1. n = 7. Data are expressed as the mean ± SD. ^*^ *p* < 0.05 *vs.* Con; ^#^ *p* < 0.05 *vs.* STZ.

As indicated in Figures 1A,B, no significant differences in basal body weight and blood glucose were observed in four groups. The body weight in the STZ group was obviously decreased compared with the Con group. The diabetic rats had the tendency of weight gain after DAP treatment, but the difference was not statistically significant. As expected, the blood glucose of the STZ rats was notably higher compared with the Con rats, and Dap treatment significantly reduced the level of blood glucose. Echocardiographic evaluation revealed that the STZ rats developed less cardiac dysfunction in response to Dap treatment as was indicated by higher LV ejection fraction (Figures 1C,D). The mRNA level of NHE-1 in the STZ group was enhanced whereas that was obviously declined after DAP treatment (Figure 1E).

### Effects of Dap on Myocardial Fibrosis, Apoptosis and Oxidative Stress in STZ-Induced Diabetic Rats

**FIGURE 2:**
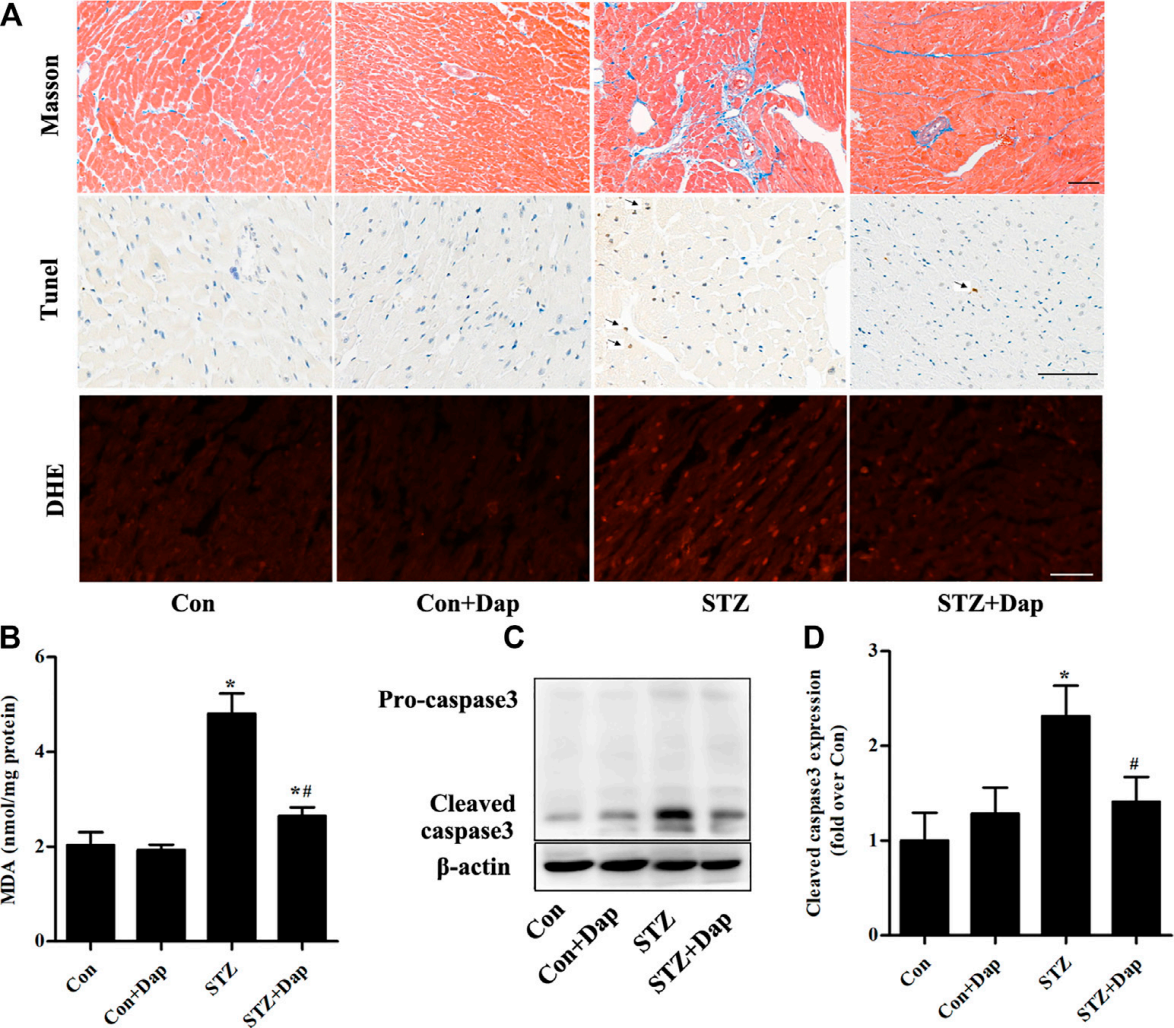
Effects of DAP on myocardial fibrosis, apoptosis and oxidative stress in diabetic rats **(A)** Upper panel, Masson’s trichrome staining of myocardium sections. Middle panel, TUNEL detection of apoptotic cells in the myocardium. Arrows point to TUNEL positive cells. Lower panel, heart sections were incubated with dihydroethidium (DHE) to evaluate superoxide anion. Scale bar = 100 µm **(B)** Myocardial malondialdehyde (MDA) concentration was assessed. *n* = 7 **(C,D)** Panel and histogram represent the expression of cleaved caspase 3 in myocardial tissues of rats. *n* = 3–4. Data are expressed as the mean ± SD. ^*^ *p* < 0.05 *vs.* Con; ^#^ *p* < 0.05 *vs.* STZ.

The Masson’s trichrome staining was measured for purpose of detecting the myocardial fibrosis degree of rats, and results demonstrated that the STZ rats had excess collagen matrix accumulation in the LV tissues. Treatment with Dap significantly ameliorated the myocardial fibrosis in STZ rats (Figure 2A). Compared with the Con rats, TUNEL-positive cardiomyocytes were increased in the heart tissues of diabetic rats, whereas those were remarkedly reduced in the Dap-treated STZ rats (Figure 2A). DHE was used to evaluate superoxide anion in the LV tissues and the content of superoxide anion in the STZ rat myocardium was obviously higher than that in the Con rats. The cardiac superoxide anion content was decreased after treatment with Dap (Figure 2A). Moreover, an increase of cardiac MDA level confirmed that oxidative damage had been induced in the STZ rats. The content of MDA in the Dap-treated STZ rats was notably reduced (Figure 2B). In addition, immunoblotting analysis showed the expression of cleaved caspase 3 protein was elevated in the heart of STZ rats. Treatment with Dap markedly decreased the myocardial cleaved caspase 3 expression (Figures 2C,D).

### Effects of DAP on Myocardial NADPH Oxidases and Antioxidant Enzymes in STZ-Induced Diabetic Rats

**FIGURE 3:**
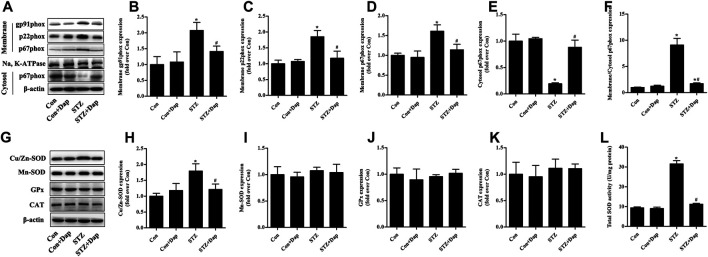
Effects of DAP on myocardial NADPH oxidases and antioxidant enzymes in diabetic rats **(A)** Panel shows representative bands of NADPH oxidases. Histograms represent densitometry analysis of the membrane gp91phox **(B)**, p22phox **(C)**, and p67phox **(D)** protein, the cytosol p67phox **(E)** protein, and the ratio of p67phox protein expression in membrane to cytosol **(F) (G)** Panel shows representative bands of antioxidant enzymes. Histograms represent densitometry analysis of the copper, zinc superoxide dismutase (Cu/Zn-SOD, **H**), manganese superoxide dismutase (Mn-SOD, **I**), glutathione peroxidase (GPx, **J**), catalase (CAT, **K**) **(L)** Myocardial levels of total SOD activity were measured. *n* = 3–4. Data are expressed as the mean ± SD. ^*^ *p* < 0.05 *vs.* Con; ^#^ *p* < 0.05 *vs.* STZ.

As mentioned above, NADPH oxidases are major origin of ROS in the heart tissues (Hansen et al., 2018; Lu et al., 2020). We measured the myocardial protein levels of NADPH oxidase subunits gp91phox, p22phox, and p67phox. Compared to the Con rats, the expression of gp91phox and p22phox in membrane fraction of myocardial tissue was up-regulated in the STZ rats, while Dap treatment down-regulated the gp91phox and p22phox expression in the diabetic rats (Figures 3A–C). Consistently, the expression of p67phox in the STZ rat heart tissues was significantly increased in membrane fraction while sharply reduced in cytosolic fraction, namely the ratio of p67phox level in membrane to cytosol which was significantly increased in diabatic rats. Dap treatment inhibited p67phox translocation to cardiac myocytes membrane in the STZ rats (Figures 3A,D–F).

As displayed in Figure 3G–L, the myocardial protein expression of Cu/Zn-SOD and total SOD activity were higher in the STZ group than those in the Con group. Dap treatment evidently reduced the elevated Cu/Zn-SOD expression and T-SOD activity in the heart tissues of STZ rats. However, there was no observed difference in the protein expression of Mn-SOD, GPx, and CAT among all experimental groups.

### Effects of DAP on High Glucose-Induced Cell Death of H9C2 Cells

**FIGURE 4:**
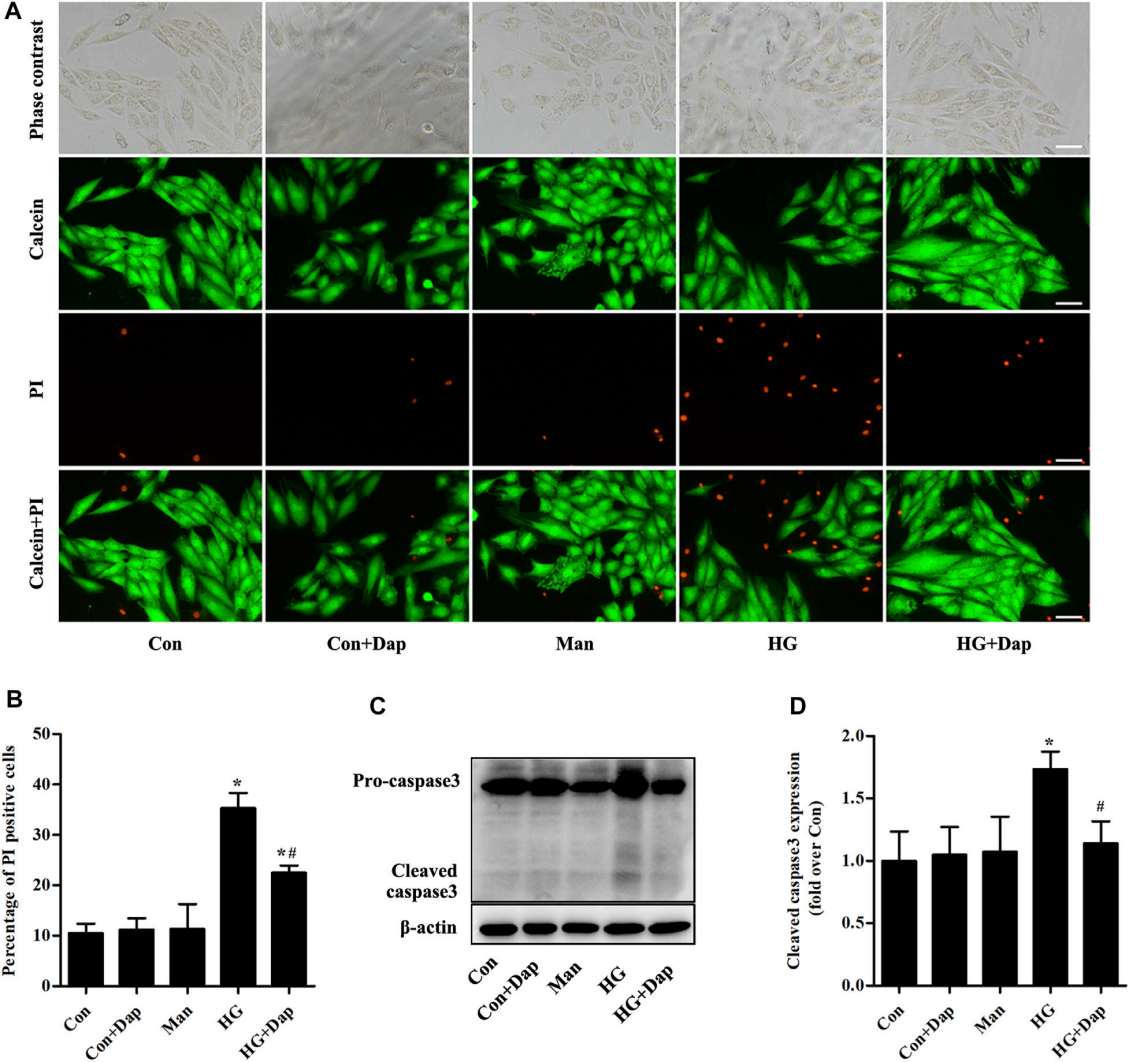
Effects of DAP on high glucose-induced death of H9C2 cells. H9C2 cells were treated with 5.5 mM glucose (Con), 5.5 mM glucose plus 10 µM Dap (Con + Dap), 33.0 mM mannitol (Man), 33.0 mM glucose (high glucose, HG) or 33.0 mM glucose plus 10 µM Dap (HG + Dap) for 36 h. Calcein acetoxymethyl ester (Calcein-AM)/propidium iodide (PI) staining was used to detect live/dead H9C2 cells **(A)** Panel shows representative photomicrographs of Calcein-AM/PI staining. Green fluorescence by Calcein-AM indicates living cells. Red fluorescence by PI represents dead cells. Scale bar = 100 µm **(B)** Histogram represents quantitative analysis of PI positive cells **(C,D)** Panel and histogram represent the expression of cleaved caspase 3 in H9C2 cells. n = 3–4. Data are expressed as the mean ± SD. ^*^ *p* < 0.05 *vs.* Con; ^#^ *p* < 0.05 *vs.* HG.

Calcein-AM/PI staining was used to reveal the effects of Dap on hyperglycemia-induced cardiomyocyte death. Compared to the Con cells, the cardiomyocyte mortality was remarkedly increased in H9C2 cells exposed to 33.0 mM glucose for 36 h. This increase was obviously ameliorated by Dap treatment (Figures 4A,B). Moreover, the expression of cleaved caspase 3 was obviously enhanced in high glucose-incubated H9C2 cells, and this change was decreased by Dap treatment (Figures 4C,D).

### Effects of DAP on High Glucose-Induced Oxidative Stress in H9C2 Cells

**FIGURE 5:**
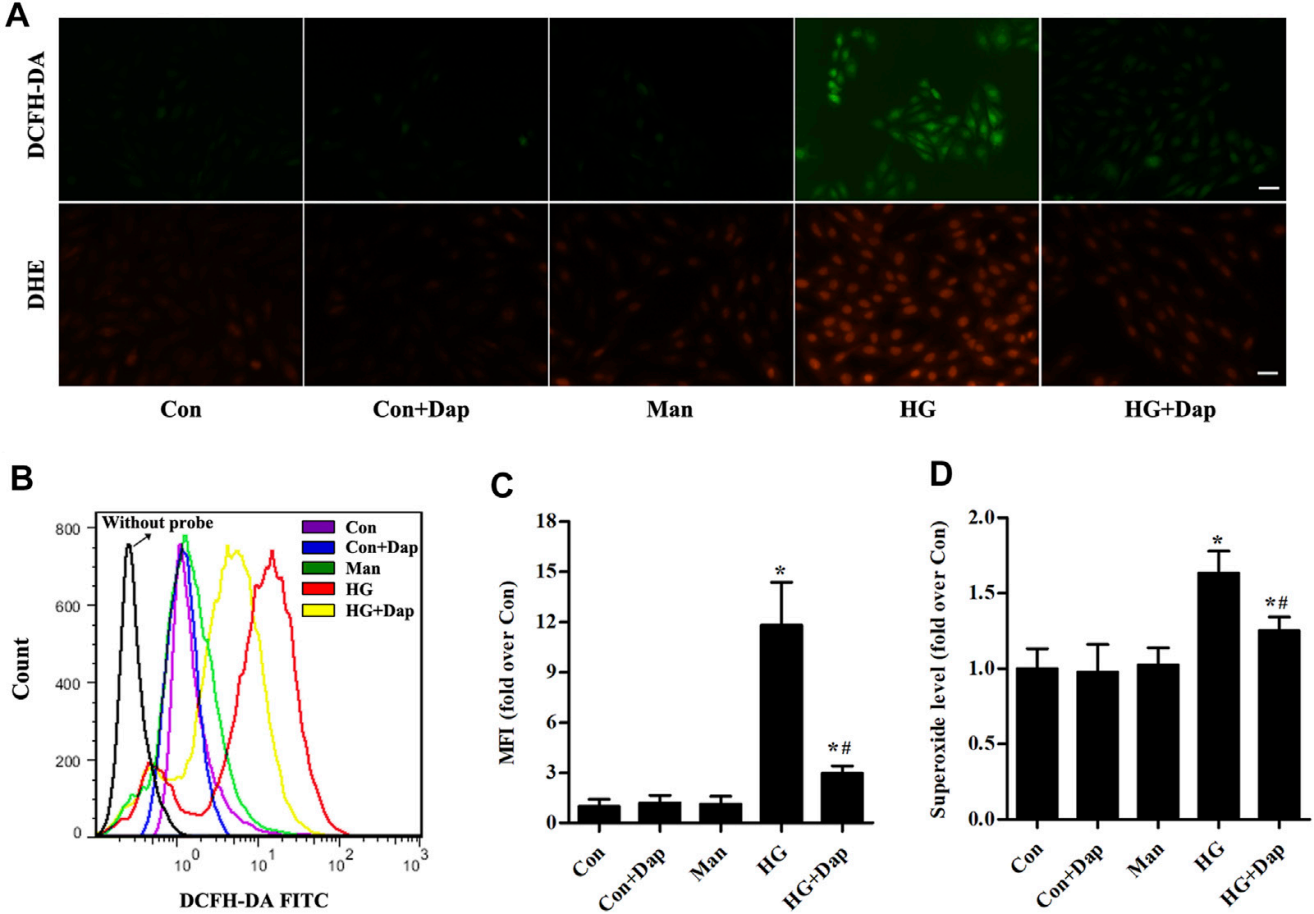
Effects of DAP on high glucose-induced oxidative stress in H9C2 cells. The treatment protocol of H9C2 cells is the same as that in Figure 4 **(A)** Upper panel, the generation of hydrogen peroxide was assessed using a DCFH-DA probe. The green signal of DCF was captured by a fluorescence microscope as presented. Lower panel, the generation of superoxide anion was assessed using a DHE probe. The red signal of ethidium was captured by a fluorescence microscope as presented. Scale bar = 100 µm **(B)** Representative pictures of ROS (hydrogen peroxide) generation in H9C2 cells measured by flow cytometry using the DCFH-DA probe **(C)** Histogram represents quantitative analysis of mean fluorescence intensity (MFI) of DCF in H9C2 cells **(D)** The levels of superoxide in H9C2 cells were assessed. n = 3–4. Data are expressed as the mean ± SD. ^*^ *p* < 0.05 *vs.* Con; ^#^ *p* < 0.05 *vs.* HG.

As shown in Figures 5A–C, the hydrogen peroxide level measured by immunofluorescence and flow cytometry analysis was significantly elevated in H9C2 cells exposed to 33.0 mM glucose for 36 h, whereas Dap treatment decreased the hydrogen peroxide production. As indicated in Figures 5A,D, the superoxide was consistently increased in high glucose-incubated H9C2 cells, and this increase was obviously reversed by Dap treatment.

### Effects of DAP on NADPH Oxidases and Antioxidant Enzymes in H9C2 Cells Incubated with High Glucose

**FIGURE 6:**
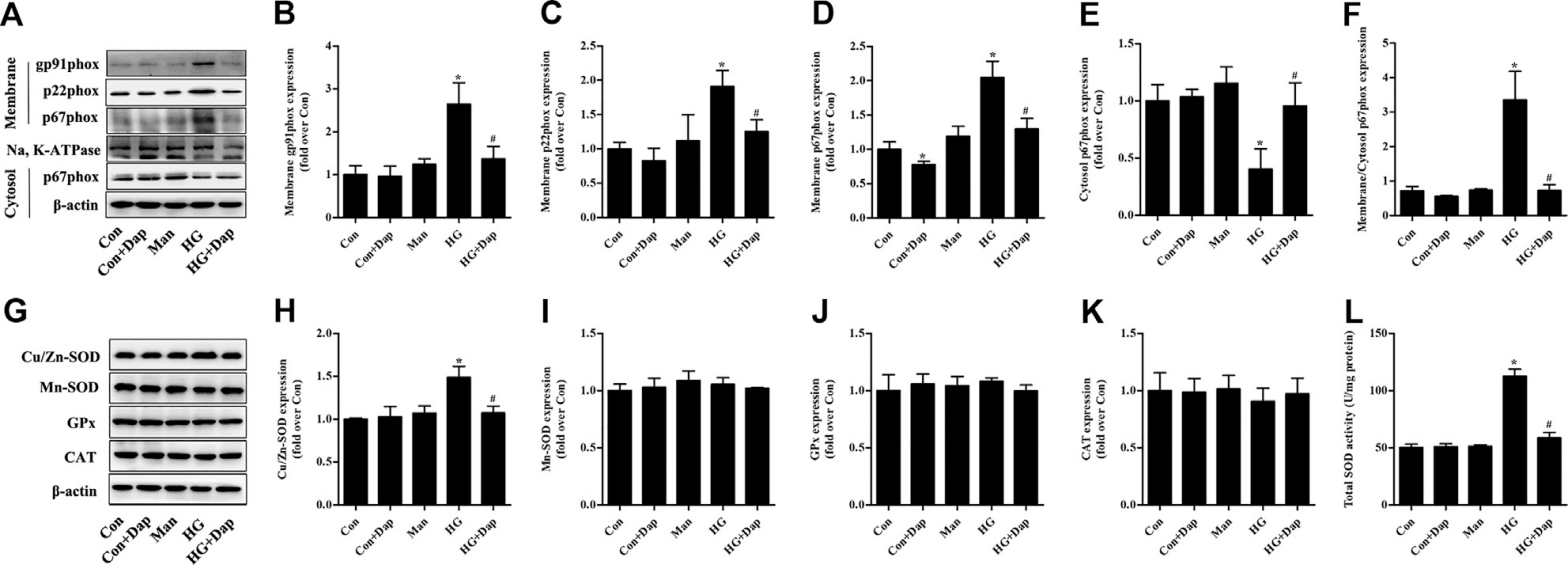
Effects of DAP on NADPH oxidases and antioxidant enzymes in high glucose-treated H9C2 cells. The treatment protocol of H9C2 cells is the same as that in Figure 4 **(A)** Panel shows representative bands of NADPH oxidases. Histograms represent densitometry analysis of the membrane gp91phox **(B)**, p22phox **(C)**, and p67phox **(D)** protein, the cytosol p67phox **(E)** protein, and the ratio of p67phox protein expression in membrane to cytosol **(F) (G)** Panel shows representative bands of antioxidant enzymes. Histograms represent densitometry analysis of the copper, zinc superoxide dismutase (Cu/Zn-SOD, **H**), manganese superoxide dismutase (Mn-SOD, **I**), glutathione peroxidase (GPx, **J**), catalase (CAT, **K**) **(L)** Levels of total SOD activity were measured. *n* = 3–4. Data are expressed as the mean ± SD. ^*^ *p* < 0.05 *vs.* Con; ^#^ *p* < 0.05 *vs.* HG.

The expression of membrane gp91phox, p22phox, and p67phox was increased in high glucose-treated H9C2 cells, while the p67phox protein level in cytosolic fraction was significantly decreased. After treatment of Dap, the expression of gp91phox and p22phox in membrane fraction as well as the ratio of p67phox expression in membrane to cytosol were obviously declined (Figures 6A–F). The expression of Cu/Zn-SOD and the activity of total SOD were elevated in high glucose-treated H9C2 cells. The protein levels of Mn-SOD, GPx, and CAT had no significant difference between each experimental group. Dap treatment significantly reduced the elevated Cu/Zn-SOD expression and T-SOD activity in high glucose-incubated H9C2 cells (Figures 6G–L).

## Discussion

Although the knowledge accumulated over the past decades, the effective treatment strategies of DCM still remain lacking. In spite of initially considered to be a hypoglycemic agent, the effects of Dap have extended far beyond that which is now being considered for improving chronic kidney disease and heart failure, even in people without DM. Similarly with the results acquired from the high fat diet and low dose STZ-induced diabetic rats, ob/ob mice, and db/db mice (Ye et al., 2017; Giuliani, 2019; Arow et al., 2020; Chen et al., 2020), the present study demonstrated that treatment with Dap for 8 weeks successfully improved cardiac dysfunction and reduced myocardial fibrosis and apoptosis in STZ-induced diabetic mice. Recent studies show that SGLT2 inhibitors increase the excretions of urinary glucose and urinary sodium in type 2 diabetic GK and OLETF rats (Chung et al., 2019; Masuda et al., 2020). The same phenomenon can be found in the Dap-treated STZ-induced diabetic rats (Chen et al., 2016), which shows that the Dap treatment may play a protective role in DCM via promoting the urinary glucose and urinary sodium excretion *in vivo*. Sodium–hydrogen exchanger (NHE) is an essential adjuster of intracellular PH and the main isoform in the heart tissues is NHE-1 (Karmazyn, 2013). There has been reported that the expression of NHE-1 was enhanced in cardiomyocytes exposed to high glucose and cariporide, a NHE inhibitor, could ameliorate the high glucose-induced myocardial hypertrophy (Chen et al., 2007). Meanwhile, NHE-1 activity was increased in the GK rats and the inhibition of NHE-1 evidently attenuated diabetic LV myocyte hypertrophy in the heart tissues of GK rats (Darmellah, et al., 2007). As shown in our study, Dap treatment declined the mRNA level of NHE-1 compared with STZ group, reflecting that the mechanism of Dap treatment improving the cardiac dysfunction might be through inhibiting the cardiac NHE-1. Furthermore, Dap incubation remarkedly reduced the mortality of H9C2 cells treated with high glucose. Our data indicate that Dap alleviates the high glucose-induced myocardial damage both *in vivo* and *in vitro*.

A large volume of evidence suggests that the enhanced ROS levels accompanied with excitation of multiple downstream pro-inflammatory and cell death pathways occupied a key position in the development of DCM (Frustaci, 2000; Westermann et al., 2007; Rajesh et al., 2010). MDA determination (a product of lipid peroxidation), superoxide anion fluorescent probe (DHE) and hydrogen peroxide fluorescent probe (DCFH-DA) were used in this study to reflect the myocardial oxidative stress status. Consistently with previous reports (Guo et al., 2007; Tsai et al., 2012; Atale et al., 2013), accumulation of ROS was detected in the LV tissues of STZ-diabetic rats and high glucose-incubated H9C2 cells. Administration of Dap for 8 weeks significantly decreased the MDA level and relieved the superoxide anion generation in heart tissues of STZ rats. However, glucose-lowering per se and other properties might contribute to the myocardial anti-oxidant stress effects of Dap. SGLT1 is the primary isoform expressed in the cardiomyocytes and there is little evidence for the expression of SGLT2 in cardiac muscle, namely Dap does not lead to reduction of glucose influx into the cardiomyocytes (Gallo et al., 2015; Oh et al., 2019). Therefore, further *in vitro* experiments were performed in cultured cardiac H9C2 cells to address the direct effects of Dap on ROS generation. As revealed by flow cytometric analyses and microscopy observations, Dap treatment markedly decreased the levels of hydrogen peroxide and superoxide anion in H9C2 cells induced by high sugar. These findings manifest the cardioprotection of Dap may be attributed, at least in part, to the direct inhibition of ROS production.

Compared with xanthine oxidases and mitochondrial respiration, NADPH oxidases have been proven to be an importance origin of ROS in heart tissues (MacCarthy et al., 2001; Hansen et al., 2018; Lu et al., 2020). Recently, it’s reported that another SGLT2 inhibitor empagliflozin ameliorates the microvascular injury of DCM by regulating AMPK signaling pathways to inhibit the mitochondrial fission (Zhou et al., 2018). Li et al. (2019) discovered that empagliflozin reduced the NADPH oxidase isoform NOX4 expression in the heart tissues of KK-Ay mice. However, the *in vivo* and *in vitro* effects of Dap on the myocardial NADPH oxidases were not addressed. Among the isoforms of NADPH oxidases, the most critical ones during the process of regulating cardiac function are gp91phox and NOX4 (Hansen et al., 2018). Interestingly, these two isoforms which mainly express in the heart have different physiological and pathophysiological effects. In the setting of sustained stress, gp91phox-dependent signaling promotes cardiomyocyte hypertrophy, contractile dysfunction, interstitial fibrosis and cell death as reviewed by Zhang et al. (2013). Contrastly, NOX4 has been attested to have protective effects, for instance, adaptively remodeling with better preserved function and improving hypertrophy following chronic hemodynamic stress (Zhang et al., 2010; Matsushima et al., 2013). Therefore, we specifically assessed the effects of Dap on changes of gp91phox isoform, p22phox subunit (regulating the stability of NADPH oxidases) and p67phox subunit (regulating the activation of NADPH oxidases) in the diabetic rats and H9C2 cells. The main new findings of the current study were that Dap treatment reduced the membrane gp91phox and p22phox expression and restrained translocation of the p67phox subunit to the membrane fraction both in STZ-induced DCM rats and H9C2 cells exposed to high glucose. Therefore, mechanisms of NADPH oxidase restraint by Dap may be the suppression of cytosolic p67phox recruitment and decrease expression of its anchor p22phox, which result in inhibiting the activation of gp91phox isoform.

SOD, GPx, and CAT are significant parts of the antioxidant defense system. Overproduction of ROS, if not controlled by these enzymes, can cause oxidative stress. The present study was designed to assess the *in vivo and in vitro* effects of Dap on the myocardial antioxidant enzymes. Similar with previous studies (Guo et al., 2007; Lei et al., 2012), the activity of T-SOD and the expression of Cu/Zn-SOD were obviously enhanced in the STZ rat myocardial tissues and high glucose-treated H9C2 cells, while protein expression of Mn-SOD, GPx, and CAT were not altered. SOD participates in the removal of superoxide anion and plays key roles in counterpoising the generation of ROS. The up-regulation of SOD might be a compensatory response when superoxide anion originated from NADPH oxidases was elevated. The increase production of superoxide anion may highly outnumber that of antioxidant enzyme SOD. Consequently, the enhanced expression and activity of SOD in the STZ rat myocardial tissues and high glucose-treated H9C2 cells do not entirely prevent but may delay the progress of cardiac oxidative damage. Dap treatment did not change the expression of Mn-SOD, GPx and CAT but evidently declined the compensatory elevated T-SOD activity and the expression of Cu/Zn-SOD. Although SOD was decreased after administration with Dap, the production of superoxide anion was greatly depressed by Dap treatment, bringing about the correction of oxidant and antioxidant imbalance and improvement of myocardial oxidative stress.

There are some limitations of our study. The cell type investigated *in vitro* is H9C2 cell, which is easy to obtain. However, it’s a rat cardiomyoblast cell line, not a cardiomyocyte. In our study, the reasons why we chose the H9C2 cell line are as follows. On the one hand, compared with neonatal rat cardiomyocyte, H9C2 cell culture is more convenient and the phenotype of H9C2 cell is more stable. On the other hand, it has been reported that the transporting system of glucose in H9C2 cells has no difference with neonatal rat cardiomyocyte and this cell line may be useful as a model for cardiocytes in aspects of transmembrane signal transduction (Hescheler et al., 1991; Yu, 1999; Yu, 2000). In addition, there are many signaling pathways of oxidative stress which play essential roles in the Dap treatment of DCM. The further underlying mechanisms how Dap influences NADPH oxidases and antioxidant enzymes need to be explored in subsequent experiment.

**FIGURE 7:**
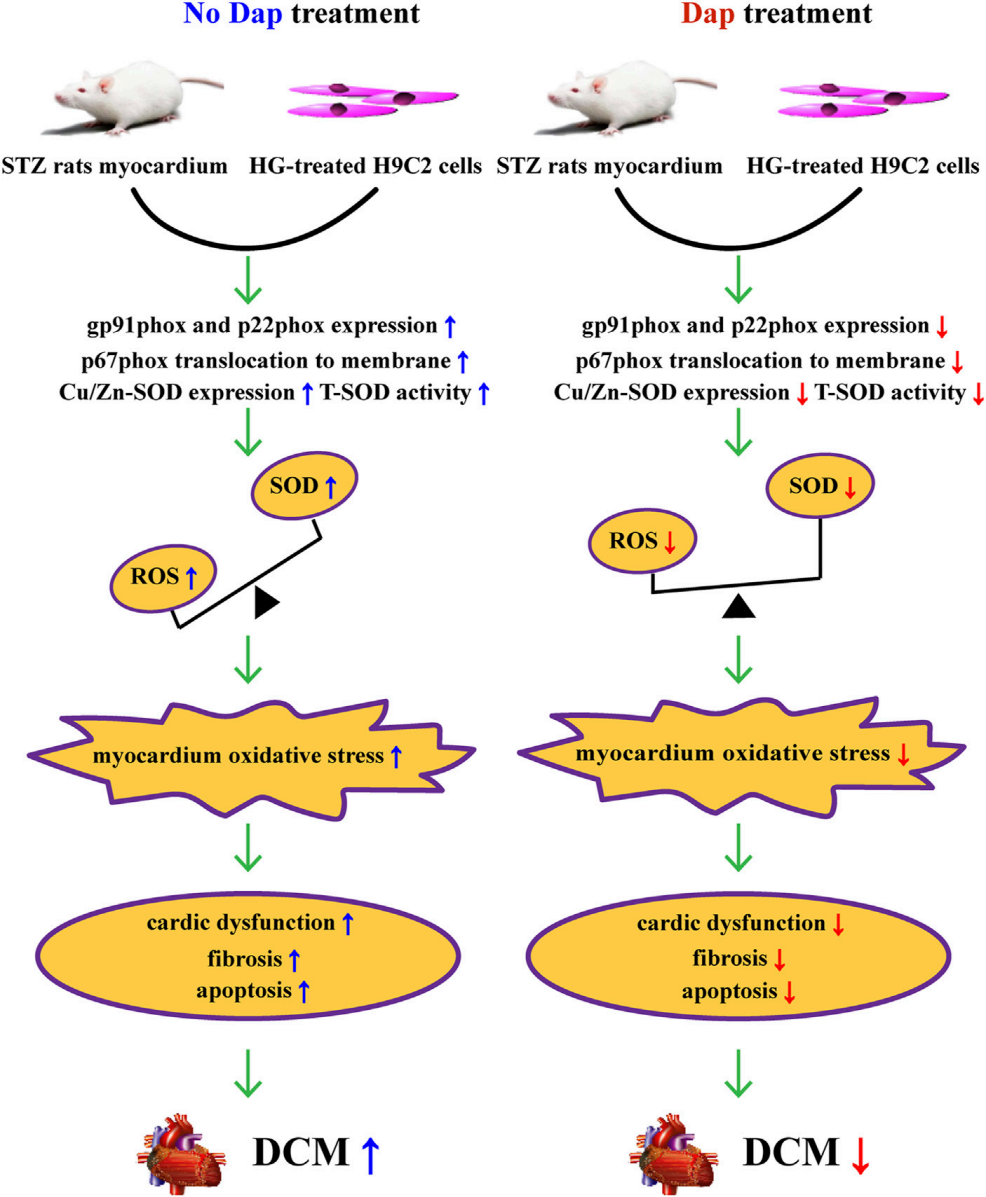
Summary of the mechanisms of Dap that suppress high glucose-induced oxidative stress *in vivo* and *in vitro*.

In summary, the cardioprotective effects of DAP in previous evidence is confirmed and extended in this study. These effects seem to be related to improvement of high glucose-induced cardiac dysfunction, myocardial fibrosis, cell death and oxidase stress, resulting from suppressing the activation of gp91phox isoform and correcting the myocardial imbalance of oxidant and antioxidant (Figure 7). Collectively, our results strongly indicate that Dap may be a potential therapeutic approach for the treatment of DCM.

## Data Availability

The original contributions presented in the study are included in the article/Supplementary Material, further inquiries can be directed to the corresponding authors.
